# Supplementary material for: Automated Synthesis of [18F]Flumazenil Application in GABAA Receptor Neuroimaging Availability for Rat Model of Anxiety
Source: Pharmaceuticals (Basel). 2023 Mar 9;16(3):417. doi: 10.3390/ph16030417 (PMC10058208; doi:10.3390/ph16030417)
Supplement: Supplementary file 1 [file pharmaceuticals-16-00417-s001.zip › pharmaceuticals-2220254-supplementary.pdf]

Table S1. The behavioral study of freezing behavior between the closed arm of 1 and 2 using different foot-shock (0.6 and 1.2 mA) and repeat times

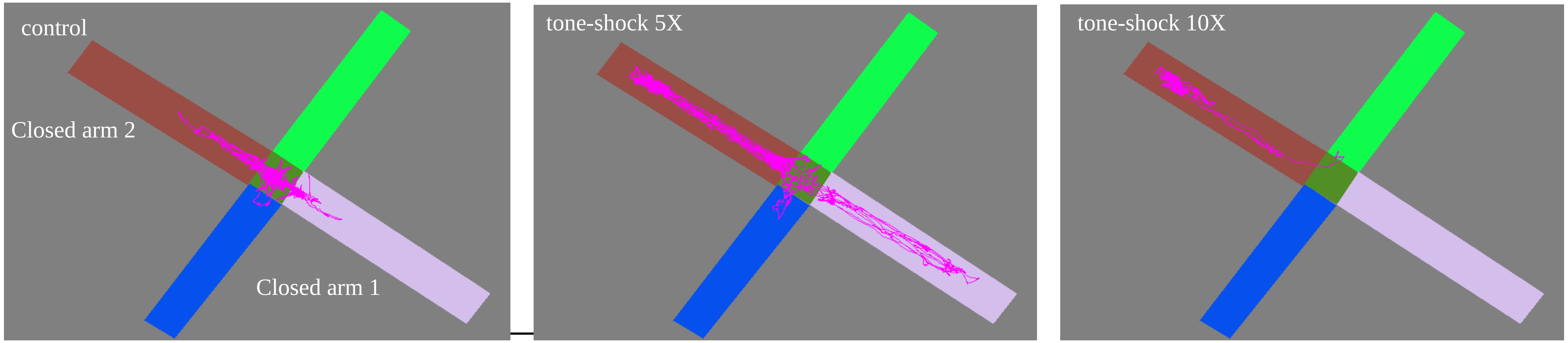

Freeze (%)	Mean ± SD
**Control group**	**26.90 ± 1.37%**
**0.6mA shock 5x**	**87.19 ± 5.78%**
**0.6mA shock 10x**	**89.01 ± 3.53%**
**1.2mA shock 10x**	**96.83 ± 1.34%**
